# Minimalistic ice recrystallisation inhibitors based on phenylalanine†

**DOI:** 10.1039/d2cc02531k

**Published:** 2022-06-10

**Authors:** Matthew T. Warren, Iain Galpin, Muhammad Hasan, Steven A. Hindmarsh, John D. Padrnos, Charlotte Edwards-Gayle, Robert T. Mathers, Dave J. Adams, Gabriele C. Sosso, Matthew I. Gibson

**Affiliations:** Department of Chemistry, University of Warwick CV5 6NP UK m.i.gibson@warwick.ac.uk; Warwick Medical School, University of Warwick CV5 6NP UK; Department of Physics, University of Warwick CV56NP UK; Department of Chemistry, Penn State University New Kensington PA 15068 USA; Diamond Light Source, Harwell Science and Innovation Campus OX11 0QX UK; School of Chemistry, University of Glasgow G12 8QQ UK

## Abstract

Ice recrystallisation inhibition (IRI) is typically associated with ice binding proteins, but polymers and other mimetics are emerging. Here we identify phenylalanine as a minimalistic, yet potent, small-molecule IRI capable of inhibiting ice growth at just 1 mg mL^−1^. Facial amphiphilicity is shown to be a crucial structural feature, with *para*-substituents enhancing (hydrophobic) or decreasing (hydrophilic) IRI activity. Both amino and acid groups were found to be essential. Solution-phase self-assembly of Phenylalanine was not observed, but the role of self-assembly at the ice/water interface could not be ruled out as a contributing factor.

## Abstract 2

Phenylalanine is demonstrated to inhibit ice recrystallisation at low mM concentrations. Sequential modifications demonstrate the importance of amphiphilicity on activity.

## Graphical abstract

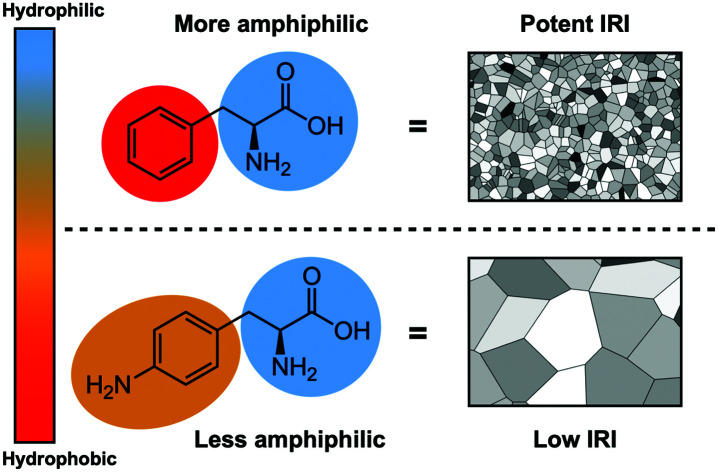

Ice binding proteins (IBPs), which include antifreeze proteins (AFPs) and ice-nucleating proteins (INPs), have evolved to control ice formation and growth.^1^ Molecules or materials which can modulate ice growth^2,3^ have potential applications in frozen food,^4^ cryopreservation^5,6^ and in transport or energy infrastructure.^7^ For many of these materials, increased molecular weight leads to higher ice recrystallisation inhibition (IRI) activity, demonstrated by the antifreeze glycoproteins (which have a repetitive tripeptide structure)^8^ and poly(vinyl alcohol),^9^ although ice-binding peptides with just 14 amino acids have been discovered.^10^ Recent evidence suggests that the assembly of AFPs into larger structures can also lead to INP-type activity.^11,12^

Despite this link between macroscopic activity and molecular size, there is emerging evidence that small(er) molecules can also have IRI activity. Ben *et al.* have explored carbohydrate derivatives,^13,14^ showing that small molecules can induce the macroscopic effect of IRI and the molecular-level mechanism may be distinct from the IBPs/polymers.^15^ Zirconium acetate can inhibit ice growth,^16^ which may be due to formation of extended structures.^17^

A common (but not exclusive) feature of many IRIs is facial amphiphilicity where hydrophilic and hydrophobic domains are segregated. For example, rigid poly(norbornene)s only inhibit ice growth when a hydrophobic face is present,^18^ hydrophobic modifications to galactose increase IRI activity,^13^ and patchy hydrophobicity has been reported.^19–21^ Safranin-O is a small molecule ice growth inhibitor,^22^ where the activity is linked to self-assembly into larger structures. Warren *et al.* recently reported that the amino acid l-α-alanine could slow ice growth, but that the isomeric β-alanine could not.^23^ Molecular simulations suggested that this was not due to ice-binding affinity, but rather differences in the compatibility with the ice lattice and ability to become overgrown by the ice. This presents the exciting possibility for the use of amino acids, and their (easily available) derivatives, as scaffolds for minimal ice growth inhibitors. Small peptides have been used as minimal^24^ protein mimics for gels and enzymes and hence are ideal targets as minimal IBP mimetics.

Here we demonstrate that l-phenylalanine (Phe) derivatives are potent IRIs capable of inhibiting ice growth at concentrations as low as 1 mg mL^−1^. Sequential modifications reveal that the amine/acid groups are crucial and that the *para*-position of the phenyl ring can be used to modulate activity. Self-assembly could be seen in the solid state, but not in solution suggesting that is not crucial for activity.

To evaluate if potent amino acid IRIs could be discovered, we tested the panel of amino acids shown in Fig. 1a. IRI activity was determined by the ‘splat’ assay (see ESI†), Fig. 1b. This assay requires some saline to avoid false positives^25,26^ and hence this screening was conducted in 10 mM NaCl.^27^ [Note, activity was reduced when phosphate ions were used]. Smaller mean grain size (MGS) indicates smaller ice crystals and hence greater inhibition activity. To assess hydrophobicity, we employed octanol-water partition coefficients (log *P*) and surface area (SA) normalised log *P* values.^28^ In Fig. 1, both log *P* and log *P*/SA values indicate increasing the hydrophobicity of the side chain from a methyl group on l-alanine to an isobutyl group on l-leucine and l-isoleucine decreased MGS values (*i.e.* more IRI activity). Furthermore, Phe was discovered to be very potent stopping all growth at just 20 mM. Log *P* (partition coefficient) and surface area (SA) normalised log *P* values^28^ for each amino acid are also shown in Fig. 1a, revealing a clear trend between increasing log *P* (more hydrophobic) and activity. l-Threonine controls resulted in larger MGS, supporting the need for hydrophobic side chains (see ESI†). d and d/l Phe controls also showed identical activity (see ESI†). The role of hydrophobicity in carbohydrate-based IRIs has been previously described.^14,29^

**Fig. 1:**
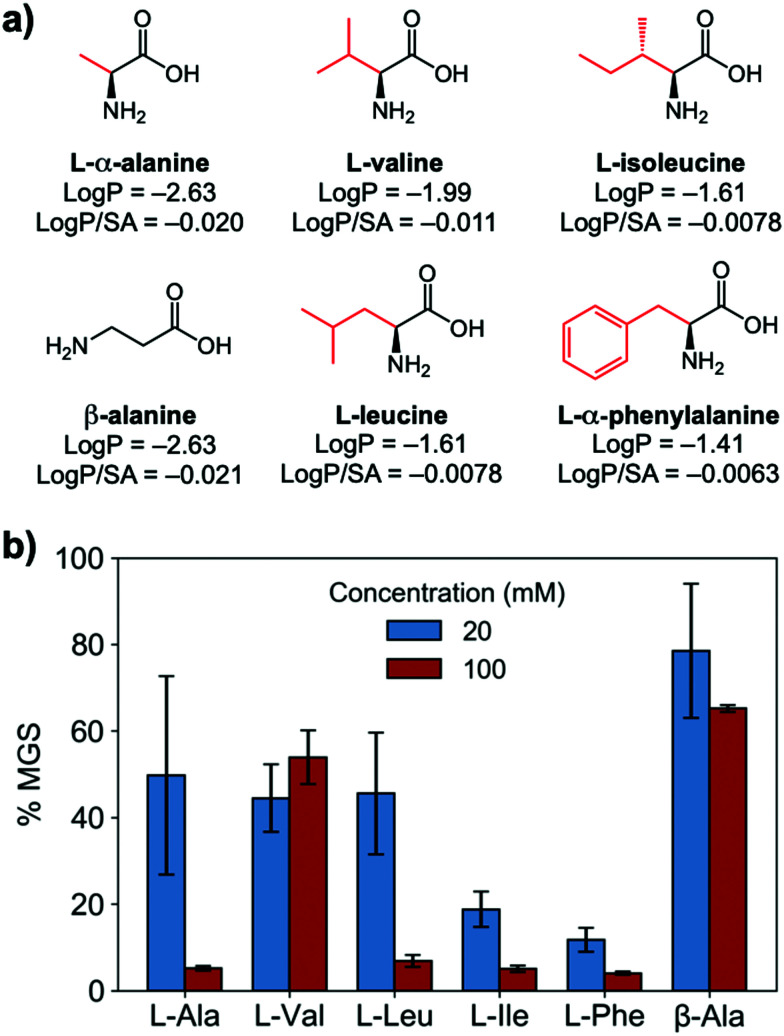
IRI activity of amino acids. (a) Chemical structures and calculated log *P* and surface area normalised log *P*; (b) IRI activity of amino acids. *n* = 3, +/− S.D. MGS = mean grain size.

A key advantage of using this amino acid platform to discover new IRIs is the accessibility (commercial/synthetic) of amino acid derivatives to explore structure-function relationships, not (easily) possible with other inhibitors. Fig. 2a shows the IRI activity of a range of *para*-modified Phe derivatives tested at 20 mM. Addition of *para*-amino or -cyano motifs lead to a significant reduction in activity (MGS > 50%). Example ice wafers to highlight this dramatic effect are shown in Fig. 2a. Halogenation (Cl/F) was a tolerated substitution with the IRI activity retained. Dose-dependency was explored for these latter modifications (Fig. 2b). Fluorination lead to a small decrease in activity, but –H or –Cl substituents both allowed potent IRI at just 10 mM, with Phe-Cl retaining activity at 5 mM which is less that 1 mg mL^−1^. These observations support a hypothesis that the hydrophobic face is essential for activity, with mean grain sizes correlating well with log *P* (ESI†). To further explore the key motifs in Phe, a panel of other derivatives were tested (Fig. 3). Switching from Phe to phenylglycine retained IRI activity, but a catechol (di-hydroxy) Phe had reduced activity. All modifications to the amine/acid face also resulted in a loss of activity. This confirms that the amine/acid are crucial for activity, which is in agreement with previous computational data for α/β-alanine, which suggests the amine/acid contacts the growing ice face, rather than the hydrophobic domain.^23^ Altogether, the data shown support a hypothesis that facial amphiphilicity is an essential motif in the activity of these amino acids, which agrees with observations of modified glycans^13^ and polymers.^18^

**Fig. 2:**
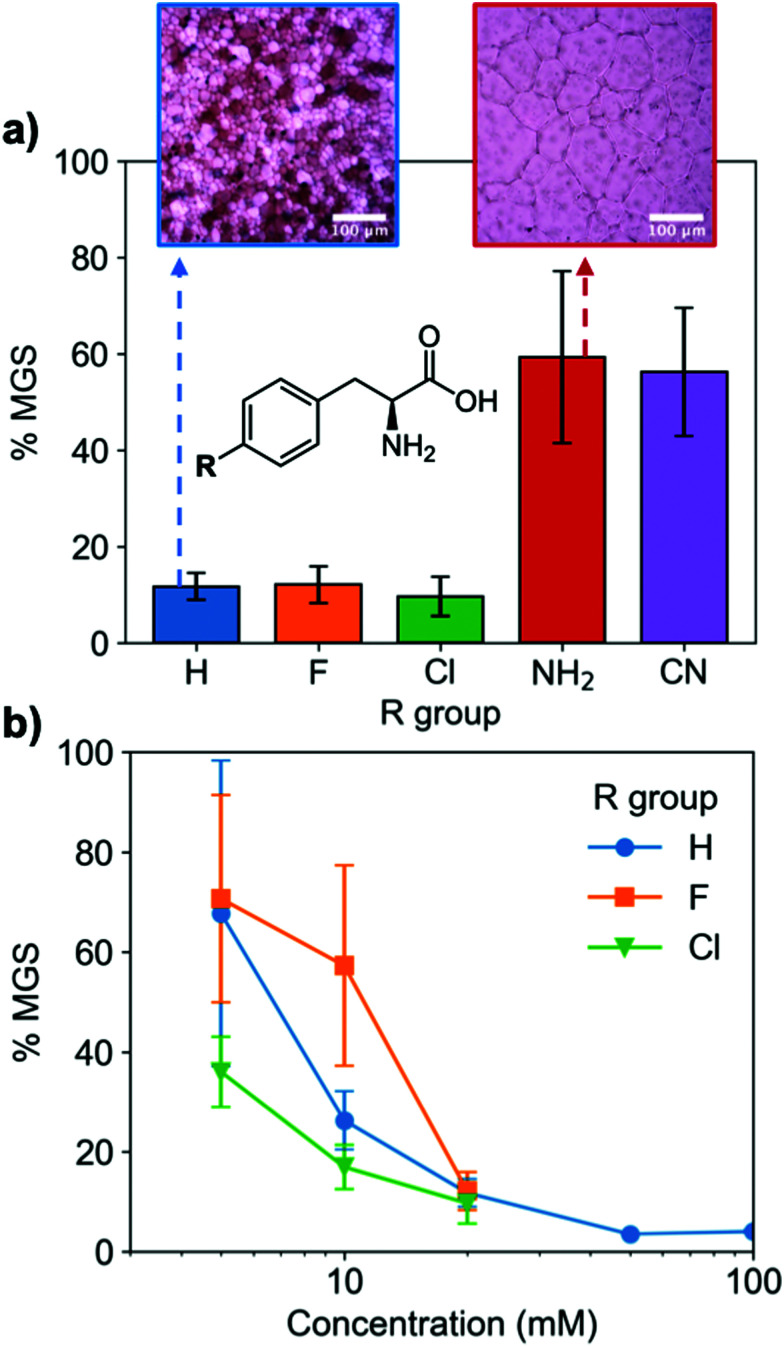
IRI activity of l-phenylalanine derivatives. (a) IRI of para-modifications at 20 mM; Inset: Example cryomicroscopy images of ice crystals annealed at – 8 °C for 30 minutes with 20 mM of indicated amino acid; (b) dose-dependent IRI of halogen-modified phenylalanine. *n* = 3, +/− S.D.

**Fig. 3:**
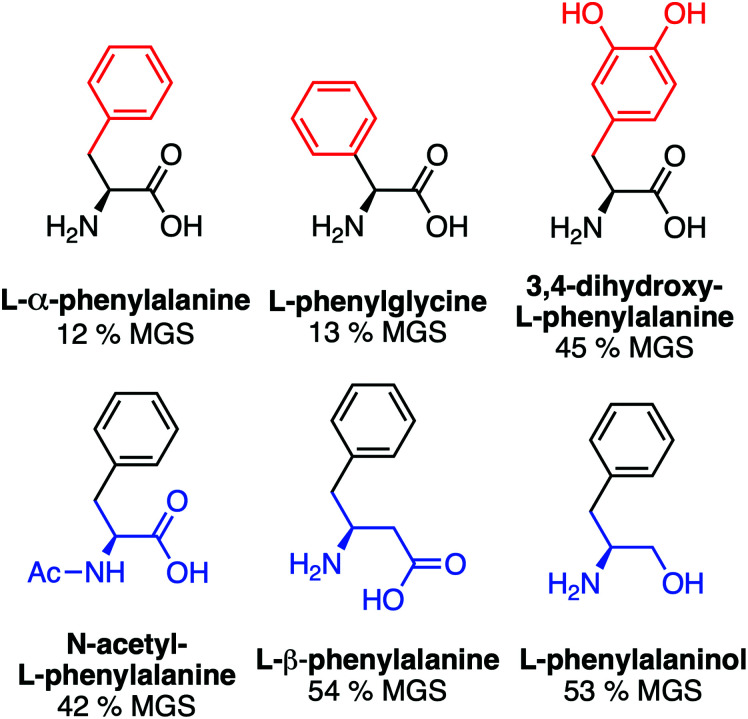
IRI activity of phenylalanine derivatives. All compounds at 20 mM in 10 mM NaCl.

The small molecule IRI-active agent safranin-O is also facially amphiphilic, but that is not the only reason for its IRI activity.^22^ The magnitude of its IRI activity was linked to its ability to self-assemble into extended 1-dimensional fibres, leading to a larger ice binding face.^22^ In contrast, Ben *et al.* have reported alkyl glycosides with IRI, which was not linked to their micellization, showing self-assembly alone is not a predictor of function.^14^ Aromatic amino acids have been reported to form amyloid-like fibrils,^30^ including the phenylalanine shown here to have IRI. To probe the role (or not) of self assembly, Fig. 4a shows dry-state SEM (scanning electron microscopy) images of Phe self-assembled fibres. This is in contrast to the IRI-inactive Phe-NH_2_ which does not form extended fibrillar structures, but rather ill-defined aggregates. Similarly, Phe-CN did not produce these fibres and does not significantly inhibit ice growth (more images are included in the ESI†). However, SEM only probes the dry state and hence can show the potential for assembly, but does not prove it was occurring under the conditions where IRI was seen. Confocal microscopy using Nile blue staining was attempted. Some fibres were observed but it was not possible to visualise a network, nor rule out Nile blue aggregates. Therefore, solution small angle X-ray scattering (SAXS) of both Phe (active) and Phe-NH_2_ (inactive) was undertaken at 1.5 mg mL^−1^, Fig. 4b and c. As can be seen there was no evidence for fibrillar assembly in either case, with the data best fitting to a power law. It is, however, important to note that it is not possible to rule out self-assembly occurring in the frozen ice wafers. As ice excludes other solutes, the unfrozen channels between wafers will contain elevated concentrations of the IRI active additives, which in turn may promote the self-assembly, not seen in dilute solution, and is the same for all IRI active agents.

**Fig. 4:**
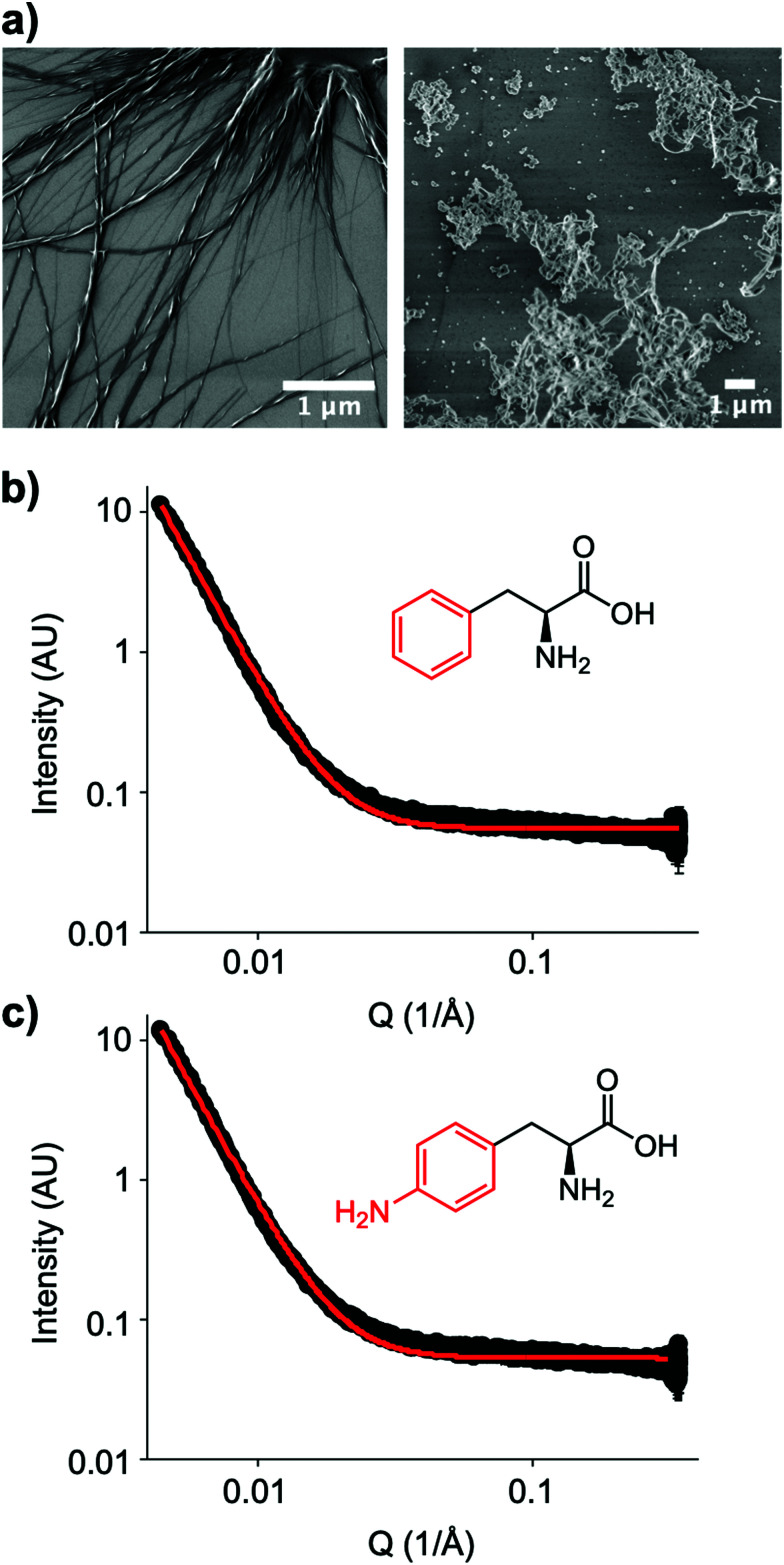
Amino acid self-assembly. (a) SEM images of l-phenylalanine (left) and 4-amino-l-phenylalanine (right) from 6 mM solutions; (b) SAXS spectra of Phe; (c) SAXS spectrum of amino-phe. SAXS conducted at 1.5 mg mL^−1^. Black line is data and red line is fit to a power law model.

These data show that the amino acid scaffolds are valuable for discovering new IRIs and understanding structure/function relationships. It is important to note, however, that for any future biomedical translation alternative inhibitors must be discovered, as Phe assembly has been linked to phenylketonuria^30^ and cytotoxicity to mammalian cell lines. High concentrations of Phe also inhibit the growth of *E.coli*.^31^

In conclusion, we have introduced phenylalanine scaffolds as a versatile small molecule tool to probe ice recrystallisation inhibition. A panel of amino acids were screened, revealing that hydrophobic side chains lead to increased IRI activity compared to hydrophilic groups. The *para* position on the phenylalanine was sequentially modified with more polar amino and cyano groups removing activity, and halogens (Cl/F) retaining all activity. It was also found that both the amino/carboxylic acid are essential for ice growth inhibition, with the modification of either group significantly reducing activity. Phe self-assembly in the dye-state was associated with activity, but it cannot be proven that the self-assembly itself was essential for activity as no assembly was seen in solution. This demonstrates that small molecule modulators of ice recrystallisation can be discovered using the simple but versatile amino acid platform. This offers opportunities to probe fundamental mechanisms through precision alteration of the chemical structure and assembly, which would be more challenging to achieve with proteins or polymeric inhibitors.

M. T. W. thanks the MRC for a studentship through the MRC Doctoral Training Partnership in Interdisciplinary Biomedical Research (MR/S502534/1). The EPSRC are thanked for a studentship to IG (EP/1938894). This project has received funding from the European Research Council (ERC) under the European Union's Horizon 2020 research and innovation programme grant agreement no. 866056. MIG thanks the Royal Society for an Industry Fellowship (191037) joint with Cytivia. We thank Max Hill for collecting the confocal images and Daniel McDowall for help collecting the SAXS data. For the purpose of open access, the author has applied a Creative Commons Attribution (CC BY) licence to any Author Accepted Manuscript version arising from this submission.

## Conflicts of interest

There are no conflicts to declare.

## Supplementary Material

CC-058-D2CC02531K-s001
