# Supplementary material for: Minimalistic ice recrystallisation inhibitors based on phenylalanine
Source: Chem Commun (Camb). 2022 Jun 10;58(55):7658–61. doi: 10.1039/d2cc02531k (PMC9260883; doi:10.1039/d2cc02531k)
Supplement: CC-058-D2CC02531K-s001 [file CC-058-D2CC02531K-s001.pdf]

Electronic Supplementary Material (ESI) for ChemComm.
This journal is © The Royal Society of Chemistry 2022

SUPPORTING INFORMATION

FOR

Minimalistic Ice Recrystallisation Inhibitors based on Phenylalanine

Matthew T. Warren,[a,b] Iain Galpin,[a] Muhammad Hasan,[a,b] Steven A. Hindmarsh,[d] John D. Padrnos,[c] Charlotte Edwards-Gayle,[f] Robert T. Mathers,[c] Dave J. Adams[e] Gabriele C. Sosso[a] and Matthew I. Gibson *[a,b]

a. Department of Chemistry, University of Warwick, UK, CV5 6NP
b. Warwick Medical School, University of Warwick, UK, CV5 6NP
c. Department of Chemistry, Penn State University, New Kensington, PA 15068, USA
d. Department of Physics, University of Warwick, UK, CV56NP
e. School of Chemistry, University of Glasgow, UK, G12 8QQ
f. Diamond Light Source, Harwell Science and Innovation Campus, UK, OX11 0QX

Email: m.i.gibson@warwick.ac.uk

Materials and Methods

L-alpha-Alanine, beta-alanine, L-isoleucine, L-leucine, L-beta-phenylalanine and 3,4-dihydroxy-L-phenylalanine were purchased from Sigma Aldrich. L-valine, L-alpha-phenylalanine, 4-amino-L-phenylalanine, 4-cyanophenylalanine, 4-chlorophenylalanine, 4-fluorophenylalanine, N-acetyl-L-phenylalanine, L-phenylalaninol and L-alpha-phenylglycine were purchased from Fischer Scientific. All amino acids were used as received. NaCl solutions were prepared using MilliQ water.

Splat cooling assay

Ice recrystallisation inhibition was measured using the "splat" cooling assay previously described by Knight et al.[1] A 10 µL aliquot of solution was dropped from a height of 1.4 m onto a glass coverslip placed on an aluminium plate cooled to −78 °C on dry ice. Upon impact with the coverslip, a polycrystalline ice monolayer approximately 10 mm in diameter and 10 µm thick is formed instantly. The coverslip was then transferred to a Linkam Cryostage BCS196 and left to anneal for 30 minutes at −8 °C. Images were obtained after 30 minutes using Canon DSLR 500D digital camera equipped to an Olympus CX 41 microscope with a UIS-2 20x/0.45/∞/0-2/FN22 lens and crossed polarisers. The mean grain size (MGS) is then determined by counting the number of crystals in the field of view (FOV) using ImageJ[2] and dividing this number by the area of the FOV. The MGS for each sample can then be compared to the average MGS for a positive control for ice growth (i.e., buffer solution only), giving a percentage MGS relative to the control (% MGS). % MGS values are reported as the average across three independent repeats.

Scanning electron microscopy

L-Phenylalanine and 4-amino-L-phenyalanine were dissolved in MilliQ water and then incubated at room temperature for 2 hours. A 4 µL aliquot of each solution was dropped onto a silicon wafer, dried under a vacuum and then coated with gold. Scanning electron microscopy images were then taken using a Zeiss GeminiSEM 500 operating between 0.5 and 2 kV.

LogP Calculations

For each amino acid, an RDKit molecule object was created from the SMILES string using the built-in function Chem.MolFromSmiles. Random conformations (~1000) were then generated using AllChem.EmbedMultipleConfs with the “useExpTorsionAnglePrefs” option enabled and the random seed set to 1. The energy of each conformation was then minimized using MMFF.GetForceField.Minimize and the conformation with the lowest energy (as given by MMFF.GetMoleculeForceField.CalcEnergy) was selected. Solvent-accessible surface area was then determined using the FreeSASA library included in RDKit (rdFreeSASA.CalcSASA), which uses a ball-rolling algorithm and Lee & Richards’ (1971) method to approximate the resulting surface area.[3] LogP values were calculated using Descriptors.MolLogP, which uses an atom-based approach.[4] All calculations were performed using RDKit version 2020.09.1.

Confocal microscopy

The samples prepared in PBS at a concentration of 6 M, with Nile Blue A added at 2 µL per mL of total solution from a stock solution of 0.1 wt%). The solutions transferred to Greiner confocal dishes (400 µL total well volume) and imaged with a Zeiss LSM 710 confocal microscope with 10x, 20x or 50x objectives. The eyepiece contains a 10x objective. Fluorescence from Nile Blue was excited using a 634 nm He-Ne laser and emission was detected between 650 and 710 nm.

Small angle X-ray scattering

SAXS measurements were performed at Diamond Light Source (Oxfordshire, UK) on the B21 beamline.[5] Samples were loaded into 1.5 mm diameter glass capillaries using a 1 mL syringe and a 19G needle. The capillaries were sealed with parafilm. The capillaries were loaded into a 3D printed cell and then loaded into the instrument via the multipurpose sample (MPS) cell.[6] 20 x 1 s frames were collected on the samples. The X-ray beam possessed a wavelength of 0.9537 Å and an energy of 13 keV. An EigerX 4M (Dectris) detector was used at a sample to detector distance of 3712.7 mm, resulting in a Q range of 0.0026-0.34 $Å^{-1}$. The data were processed in Dawn Science (version 2.25, https://dawnsci.org/). The scattering from deionised water in a glass capillary was used as the background. The 2D images were azimuthally integrated to produce the 1D I vs Q plots.

Additional Data

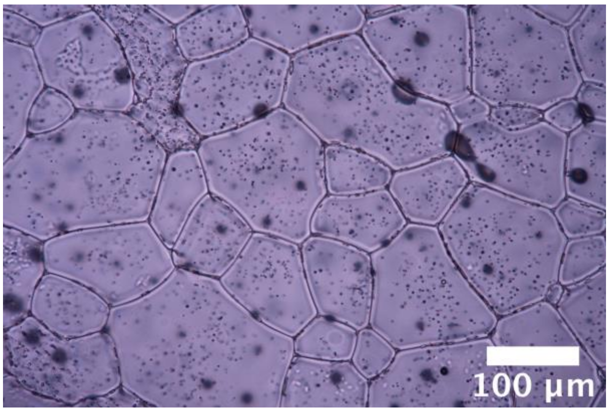

Figure S1: Example cryomicrograph from "splat"-cooling assay for 20 mM L-threonine. Measurement was performed in 10 mM NaCl solution.

Table S1: LogP values, surface areas (SA) and volumes for substituted L-phenylalanines. ΔLogP values were calculated via subtracting LogP values with LogP value for L-glycine (-3.021)

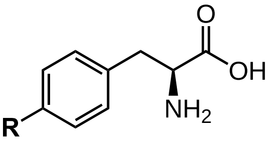

R group	LogP	SA	LogP/SA	ΔLogP	Vol	SA/Vol	MGS (%)
**H**	-1.410	225.37	-0.0063	1.611	156.64	1.439	12
**F**	-1.271	228.09	-0.0056	1.751	162.21	1.406	12
**Cl**	-0.757	229.05	-0.0033	2.265	172.20	1.330	10
**NH2**	-1.828	241.57	-0.0076	1.194	168.72	1.432	59
**CN**	-1.539	235.74	-0.0065	1.483	174.79	1.349	55

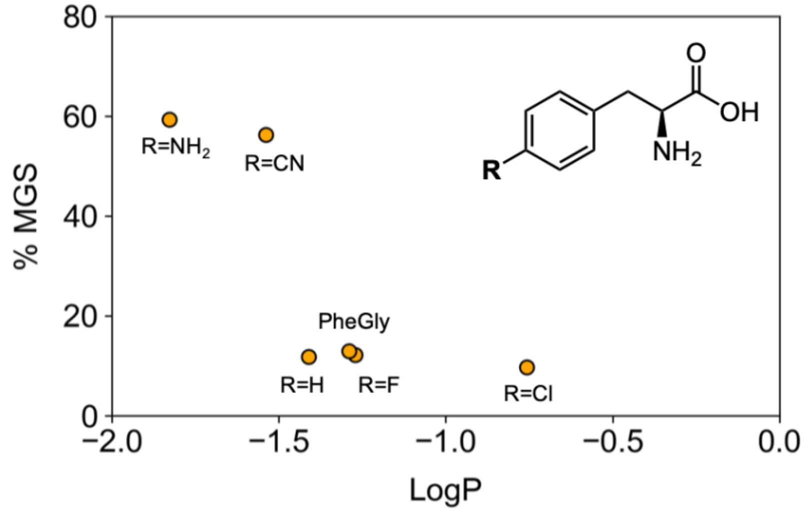

Figure S2. LogP values verses mean grain size (% MGS).

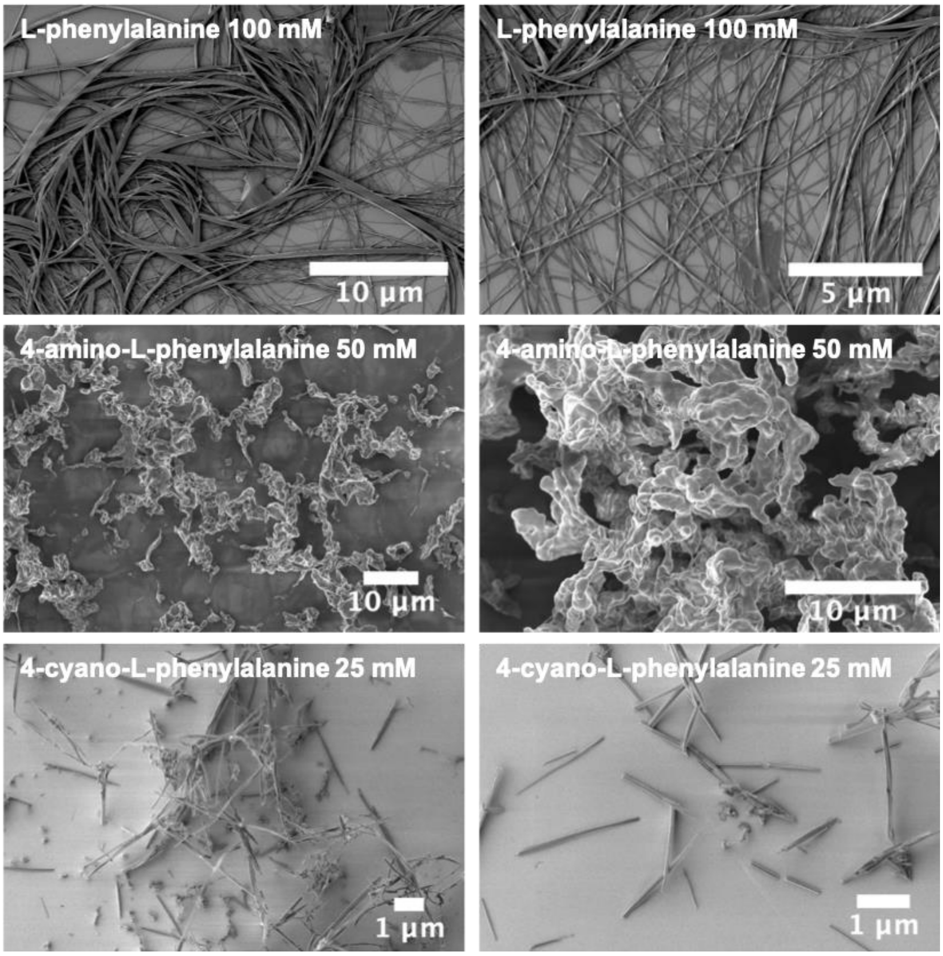

Figure S2: SEM images of L-alpha-phenylalanine, 4-amino-L-phenylalanine and 4-cyano-L-phenylalanine prepared from 100 mM, 50 mM and 25 mM solutions, respectively.

Table S2: Fit parameters for the SAXS data to a power law model.

	4-Amino-Phe	Phe
Background	$0.051 \pm 3.96x10^{-5}$	$0.053 \pm 4.25x10^{-5}$
Scale	$4.09x10^{-8} \pm 7.52x10^{-10}$	$3.99x10^{-8} \pm 7.96x10^{-10}$
Power	3.60 ± 0.004	3.59 ± 0.004
χ^2	4.9667	4.1654

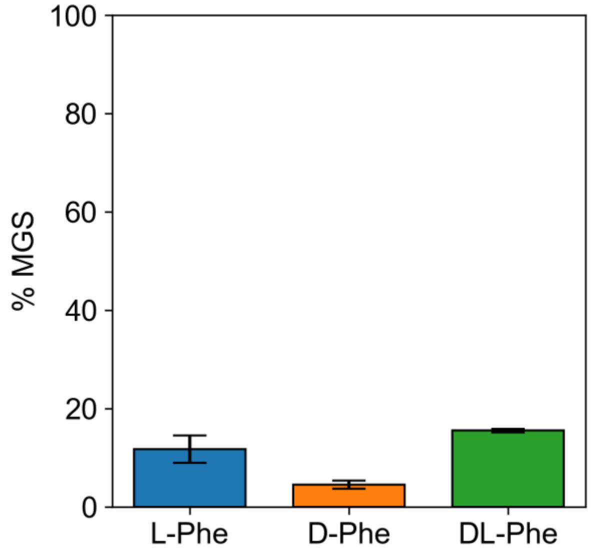

Figure S4: Ice recrystallisation inhibition activity of phenylalanine enantiomers at 20 mM. All conducted in 10 mM NaCl. Mean grain size (MGS) is reported relative to saline, following 30 minutes at – 8 °C.

References

(1) Knight, C. A.; Hallett, J.; DeVries, A. L. Solute Effects on Ice Recrystallization: An Assessment Technique. *Cryobiology* **1988**, *25* (1), 55–60.

(2) Schindelin, J.; Arganda-Carreras, I.; Frise, E.; Kaynig, V.; Longair, M.; Pietzsch, T.; Preibisch, S.; Rueden, C.; Saalfeld, S.; Schmid, B.; et al. Fiji: An Open-Source Platform for Biological-Image Analysis. *Nat. Methods* **2012**, *9* (7), 676–682.

(3) Mitternacht, S. FreeSASA: An Open Source C Library for Solvent Accessible Surface Area

Calculations. *F1000Research* **2016**, *5*, 189.

(4) Wildman, S. A.; Crippen, G. M. Prediction of Physicochemical Parameters by Atomic Contributions. *J. Chem. Inf. Comput. Sci.* **1999**, *39* (5), 868–873.

(5) Cowieson, N. P.; Edwards-Gayle, C. J. C.; Inoue, K.; Khunti, N. S.; Doutch, J.; Williams, E.; Daniels, S.; Preece, G.; Krumpa, N. A.; Sutter, J. P.; et al. Beamline B21: High-Throughput Small-Angle X-Ray Scattering at Diamond Light Source. *J. Synchrotron Radiat.* **2020**, *27* (5), 1438–1446.

(6) Edwards-Gayle, C. J. C.; Khunti, N.; Hamley, I. W.; Inoue, K.; Cowieson, N.; Rambo, R. Design of a Multipurpose Sample Cell Holder for the Diamond Light Source High-Throughput SAXS Beamline B21. *J. Synchrotron Radiat.* **2021**, *28* (1), 318–321.
